# Association of mitochondrial DNA variants with myalgic encephalomyelitis/chronic fatigue syndrome (ME/CFS) symptoms

**DOI:** 10.1186/s12967-016-1104-5

**Published:** 2016-12-20

**Authors:** Maureen R. Hanson, Zhenglong Gu, Alon Keinan, Kaixiong Ye, Arnaud Germain, Paul Billing-Ross

**Affiliations:** 1 Department of Molecular Biology and Genetics, Cornell University, Ithaca, NY 14853 USA; 2 Division of Nutritional Sciences, Cornell University, Ithaca, NY 14853 USA; 3 Department of Biological Statistics and Computational Biology, Cornell University, Ithaca, NY 14853 USA

**Keywords:** Myalgic encephalomyelitis (ME), Chronic fatigue syndrome (CFS), Next-generation sequencing, Mitochondrial DNA, mtDNA, Heteroplasmy, Association, SNPs, Haplogroup, Variants

## Abstract

Earlier this year, we described an analysis of mitochondrial DNA (mtDNA) variants in myalgic encephalomyelitis (ME)/chronic fatigue syndrome (CFS) patients and healthy controls. We reported that there was no significant association of haplogroups or singe nucleotide polymorphisms (SNPs) with disease status. Nevertheless, a commentary about our paper appeared (Finsterer and Zarrouk-Mahjoub. J Transl Med14:182, 2016) that criticized the association of mtDNA haplogroups with ME/CFS, a conclusion that was absent from our paper. The aforementioned commentary also demanded experiments that were outside of the scope of our study, ones that we had suggested as follow-up studies. Because they failed to consult a published and cited report describing the cohorts we studied, the authors also cast aspersions on the method of selection of cases for inclusion. We reiterate that we observed statistically significant association of mtDNA variants with particular symptoms and their severity, though we observed no association with disease status.

Following the publication of our paper [1] concerning mitochondrial DNA variation in ME/CFS patients and controls, Finsterer and Zarrouk-Mahjoub [2] published a commentary on our paper entitled “Is chronic fatigue syndrome truly associated with haplogroups or mtDNA single nucleotide polymorphisms?” Anyone reading this title might assume that our paper claimed a correlation of mtDNA SNPs with disease status. In fact, in describing our paper they wrote that “the study showed that CFS is associated with mtDNA haplogroups J, U and H”. However, we concluded exactly the opposite. We reported that we did not detect any statistically significant association of mtDNA variation with patient vs. control status. Instead, we reported that individuals of certain haplogroups or carrying certain mtDNA SNPs were more likely to report experiencing particular symptoms or certain severity levels of particular symptoms [1]. It is evident from the title of the commentary, as well as its content, that Finsterer and Zarrouk-Mahjoub [2] did not read our paper carefully.

The authors criticized our study for examining mtDNA variation only, rather than variation in nuclear genes encoding proteins involved in mitochondrial functions. Because the funding available allowed us only mtDNA analysis, we made no claims in the paper that all genes affecting mitochondrial performance had been analyzed. We stated clearly that we were analyzing mtDNA variation, not all genetic variation that could affect mitochondrial function. Moreover, the last sentence of our discussion sets forth the need for “further analysis of the mitochondrial and nuclear genomes.”

The authors also expressed concern that we might have missed some heteroplasmic mutations because of the use of DNA from lymphocytes, rather than from other tissues. We reported very low levels of heteroplasmy in our samples, which were comprised of DNA from whole blood of ME/CFS patients and controls. Whole blood DNA was available to us; DNA from other tissues was not. Although we were aware that tissue specificity of mtDNA heteroplasmy exists [3], it is highly unlikely that any subjects harbored physiologically significant amounts of a heteroplasmic mutation despite our use of blood DNA, which is suitable for identifying mutations in most mtDNA-encoded diseases, with one exception. It is known that individuals with MELAS, especially as age increases, sometimes harbor far fewer abnormal mtDNAs in their blood cells than in other cell types [4, 5]. Someone with severe MELAS was unlikely to remain undetected, given the number of differential symptoms that would have allowed the ME/CFS experts, who provided the samples, to identify any such individual victim. Furthermore, while in a study of 34 Dutch families, some individuals harbored undetectable levels of the MELAS mutations in blood but significant amounts in other cell types, the majority of MELAS carriers in that study did have detectable heteroplasmy in blood [6].

The authors then pointed out that we did not carry out “immune-histological nor biochemical studies of muscle biopsies” and that such studies would be valuable to identifying dysfunctional mitochondrial pathways. We do not disagree with this point, but again we made no claim that we had detected all possible such dysfunctions, given that such experiments were outside the scope of our project. While such studies might be of future interest, obtaining muscle biopsies from a patient population that usually exhibits high sensitivity to pain poses ethical considerations.

Finsterer and Zaroub-Mahjoub [2] then ask how other causes of fatigue were distinguished from ME/CFS and then imply that our subject cohort could have included individuals with a number of other diseases. Had the authors consulted the paper we cited [7], which provides a thorough discussion of how ME/CFS subjects were screened for other diseases and selected for inclusion by 6 ME/CFS expert physicians, they would find their worries about proper subject selection to be unjustified. The authors complain that “nothing is reported about the previous history of the 193 patients.” They are correct, because such information was already reported in detail in the paper authored by the ME/CFS experts [7], who have collected an extremely valuable set of samples that have been [8] and will undoubtedly be used for many other types of studies. We encourage readers of our study and other studies utilizing the Chronic Fatigue Initiative sample set to consult the foundational paper describing the cohort [7] instead of making unwarranted negative assumptions about its quality.

## Abbreviations

ME/CFS: myalgic encephalomyelitis/chronic fatigue syndrome
mtDNA: mitochondrial DNA
SNP: single nucleotide polymorphism
